# Ecotoxicity Study of New Composite Materials Based on Epoxy Matrix DER-331 Filled with Biocides Used for Industrial Applications

**DOI:** 10.3390/polym14163275

**Published:** 2022-08-11

**Authors:** Anton Panda, Kostiantyn Dyadyura, Jan Valíček, Marta Harničárová, Milena Kušnerová, Tatyana Ivakhniuk, Liudmyla Hrebenyk, Oleksandr Sapronov, Vitalii Sotsenko, Pavlo Vorobiov, Vitalii Levytskyi, Andriy Buketov, Iveta Pandová

**Affiliations:** 1 Department of Automobile and Manufacturing Technologies, Faculty of Manufacturing Technologies of the Technical University of Košice with the seat in Prešov, Bayerova 1, 080 01 Prešov, Slovakia; 2 Department of Biomedical Engineering, Institute of Medical Engineering, Odessa Polytechnic National University, Shevchenka Ave, 1, 65044 Odessa, Ukraine; 3 Department of Mechanical Engineering, Faculty of Technology, Institute of Technology and Business in České Budějovice, Okružní 10, 370 01 České Budějovice, Czech Republic or; 4 Department of Electrical Engineering, Automation, Informatics and Physics, Faculty of Engineering, Slovak University of Agriculture in Nitra, Tr. A. Hlinku 2, 949 76 Nitra, Slovakia; 5 Department of Public Health, Medical Institute, Sumy State University, 2, Rymskogo-Korsakova St., 40007 Sumy, Ukraine; 6 Department of Biophysics, Biochemistry, Pharmacology and Biomolecular Engineering, Medical Institute, Sumy State University, 2, Rymskogo-Korsakova St., 40007 Sumy, Ukraine; 7 Department of Transport Technologies, Marine Engineering Faculty, Kherson State Maritime Academy, Ushakova Ave, 20, 73000 Kherson, Ukraine; 8 Computer-Integrated Technologies Department, Faculty of Applied Information Technologies and Electrical Engineering, Ternopil Ivan Puluj National Technical University, Ruska Str., 56, 46001 Ternopil, Ukraine; 9 Department of Natural Sciences and Humanities, Faculty of Manufacturing Technologies of the Technical University of Košice with the seat in Prešov, Bayerova 1, 080 01 Prešov, Slovakia

**Keywords:** epoxy-composite, antifouling, bioaccumulation, marine pollution, structure of surface, marine bacteria, *Bifidobacterium bifidum*, *Lactobacillus acidophilus*

## Abstract

The impact of fillers in the epoxy oligomer on the test culture of the marine ecosystem was investigated. The content of additive—biocides—was selected based on the complex research using STAT-GRAPHICS^®^ Centurion XVI. The ecotoxicity of composite surfaces was determined in model systems using methods which are standard in eco-microbiology. The microorganism was identified by studying morphological, cultural, biochemical, and antigenic properties. Studies of the structure and the microrelief of the surfaces of composite materials were conducted using scanning electron microscopy (SEM) and X-ray energy dispersive spectroscopy. Based on comprehensive research, it was established that the composition of oxytetracycline with content of *q* = 1.5 wt% and nanosilver with a content of *q* = 0.075 wt% per 100 wt% of the DER-331 oligomer and 10 parts by the mass of the TETA hardener ensures the formation of a porous nano-heterogeneous structure of the coating, which leads to the acceleration of the release of silver ions from the surface of the polymer. The rational content of the complex additives of biocides ensures adhesion to the cell wall of bacteria with subsequent penetration into it and subsequent change to the cell membrane, its death, and, therefore, the suppression of the fouling process of metal structures.

## 1. Introduction

The search for ways to improve the performance of mechanisms and parts of maritime transport corresponds to the general concept of sustainable development, which involves the conservation and rational usage of marine resources and the implementation of mindful consumption and production, which contributes to better socio-natural well-being [1,2]. The preservation of marine ecosystems significantly contributes to people’s well-being and brings economic benefits that are mainly incalculable [3]. Marine ecosystems contain a wide variety of organisms that live in extreme conditions [4], and their reactions to new technological implementations must be accounted for. Although, in the literature, less attention is paid to the conservation of marine ecosystems than terrestrial ecosystems, the current biodiversity crisis requires an urgent need for research that can increase the effectiveness of the practical use of scientific results in this direction [5,6]. This concerns, first of all, the competent, environmentally friendly use of new materials that have significant economic benefits [7]. Due to the fast development of nanotechnology in recent years, scientists have been working toward the creation of new materials that, without the environmental damage, could significantly increase the profitability and productivity of the marine economy, especially related to shipping [8]. One of the approaches to increase the service life of the parts and mechanisms of maritime transport is the use of composite materials (CM) to protect surfaces from fouling [9,10]. The biological fouling of ships and structures significantly impairs the efficiency of these facilities and increases the economic costs associated with their operation, maintenance, and repair [11]. Currently, modified epoxy composites are widely used [12,13]. In a review of 322 studies published since 1978, one of the priorities for developing the antifouling composite field was identified, namely, the growing interest in the use of environmentally friendly materials [14]. At the same time, in studies of composite coatings with antifouling properties, emphasis is placed on the technical parameters of coatings, on optimizing the recipe of mixed composites according to practical objects, and on the effect of filler nanoparticles on surface structure formation [15]. Coatings based on epoxy polymers are widely used to improve the performance of technological equipment used in the construction of ships and in offshore structures [16,17]. A comprehensive review of epoxy resins’ possible applications was given in these articles [18,19]. 

At the same time, the practical use of any chemical intensifies the issues of their environmental toxicity and impact on ecosystems in the long run. Numerous studies on the bioaccumulation of anthropogenic chemical pollutants indicate that biocides have a long half-life and can accumulate in the environment [20,21,22,23,24,25,26,27]. Changes in the chemical composition of the aquatic environment threaten the species diversity of ecosystems [28,29]. According to many researchers, the uncontrolled use of anthropogenic pollutants leads to the destruction of the evolutionarily reconstructed relationships of living organisms, starting with the simplest [30,31]. In recent years, the number of studies examining the effects of anthropogenic chemical factors on the microbiota has increased [31]. 

It is a well-known fact that microorganisms are critical for modeling and studying the F and T (Fate and Transport) and the effects of engineering nanomaterials entering aquatic biocenosis. The creation of model systems for predicting the realistic fate and action of nanomaterials using microorganisms is based on existing data about the toxic effect of some nanoparticles on microscopic objects in marine systems [32,33].

Modeling the impact of nanoparticles released from composites on microorganisms allows the minimizing of their potential toxic impact on the environment during further practical implementations of antifouling coatings. The creation of composites to protect surfaces from fouling in an aquatic environment is based on the known facts of the antibacterial effect of some nanoparticles, for example, nanosilver (NS) [34]. The problem of using such nanoparticles is related to the search for a consensus between the prevention of fouling and the ecotoxicity of new nanomaterials. The modeling of the biological effects of nanoparticles using the simplest organisms makes it possible to determine the environmentally friendly optimum of the nanomaterial, which will ensure its safe, practical use.

The lack of a precise algorithm for modeling the toxic effects of chemical “intervention” on the ecosystem as a whole is effectively compensated for by studies of the initial stage of interaction between the factor and the biocenosis, which is considered a trigger for possible future negative changes in ecosystems. The study of changes in the microbiota of the aquatic environment and living organisms can serve as a source of information for predicting the biological effects of chemicals on aquatic organisms.

To study the formation of biofilms on polymer coatings with antifouling properties, the microorganism characteristics of aquatic systems are used, such as *Pseudoaltermonas tunicata*, *Pseudomonas* sp. SS304, *Cyanobium* sp. LEGE 10375, *Roseobacter*, and others [35,36]. They are early colonizers on surfaces during fouling. 

From the viewpoint of the authors of most studies, these microscopic organisms determine the biofilm’s structure and functions on artificial and natural surfaces in the aquatic environment and create conditions for attaching larger organisms. The creation and study of antifouling coatings involve the study of primary biofilms in test systems that simulate similar processes in natural conditions. Usually, under experimental conditions, monocultures of the indicated microorganisms are used. Strictly for certain species of microorganisms, unequivocal conclusions regarding the formation of biofilms can be made during the study of the action of nanoparticles of antifouling materials on the microbiota of the hydrogen environment. As is known, in natural conditions, multispecies biofilms are formed.

The main studies focus on the behavior of individual representatives of microbiota on the effect of nanoparticles. The study of the formation of multispecies biofilms has technical and theoretical issues for interpreting the results [37].

One of the promising components for forming an anti-adhesive coating is silver nanoparticles that have antibacterial properties [38,39].

Studies of biofilms are used to assess the effectiveness and toxicity of similar biocides in creating new composites. It is known that coatings with nanoparticles of silver, copper, and other metals prevent the formation of adhesive structures of microorganisms.

Simultaneously, there have not been many studies on biofilms in the natural environment and the role of microorganisms in the biodegradation of nanoparticles, and this area requires further research in the context of the growing number of new materials. Microbial biofilms on surfaces are fundamental to aquatic ecosystems and play a key role in macrofouling. On an industrial scale, they are of interest because of their adverse impact on artificial surfaces in the aquatic environment, which leads to the surface strain of hulls, microbial blistering, and the corrosion of metal surfaces. Scientists suggest that, in the future, biofilms can be used to counteract or control these processes, as well as for bioremediation of environmental pollution [40].

The study of the process of biofilm formation on modified surfaces can be helpful, both in terms of assessing the dynamics of biofouling and in terms of studying the toxic effects of new materials on the aquatic environment [41,42].

This study aimed to assess the toxicity and inhibition of biofouling using new composite polymeric material based on epoxy oligomer DER-331 with additive–biocides by studying the process of multispecies biofilm formation and analysis of antibacterial properties in model test systems of microorganisms.

## 2. Materials and Methods

### 2.1. Technology of the Composite Material Formation

Epoxy oligomer DER-331 (CAS No. 25085-99-8), manufactured by Dow Chemical Comp (Merck KGaA, Darmstadt, Germany), was used to form epoxy-composite materials. Triethylenetetramine TETA hardener (CAS No. 112-24-3, Cleveland, OH, US) was used to crosslink the epoxy composition. The content (in the weigh fraction *q*) of the hardener was *q* = 10 wt%, indicated per 100 parts by weight of epoxy resin DER-331 (Cleveland, OH, USA).

Nanosilver (NS) and (OT) oxytetracycline (Billerica, MA 01821, US) were used as biocide fillers. NS belongs to the group of metals of intermediate thermodynamic stability. Silver nanoparticles have a positive standard electrode potential, which does not exceed the electrode potential value associated with the oxidizing action of oxygen in a neutral environment. Therefore, the filler is resistant to any acidic and neutral environments without oxygen. This biocide provides high antibacterial activity against microorganisms. The dispersion of NS was 10–100 nm [43].

**Figure 1:**
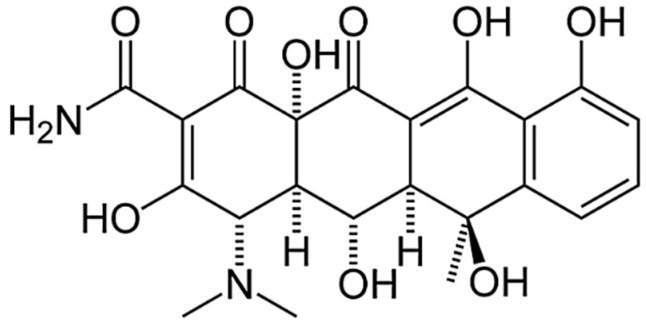
The structural formula of oxytetracycline.

A tetracycline antibiotic, oxytetracycline, was also used. It had a dispersion of 5–10 microns. This compound has various biocidal properties and can be used against many bacterial organisms. OT is, by nature, a lipophilic substance and can easily penetrate the cell membrane of bacterial microorganisms, which inhibits the development of their population. Therefore, it is advisable to use it as an antibacterial ingredient in the formation of the epoxy composition, which will prevent fouling of the hull by microorganisms. The molecular formula of this filler is C_22_H_24_N_2_O_9_. The structural formula is shown in Figure 1.

**Figure 2:**
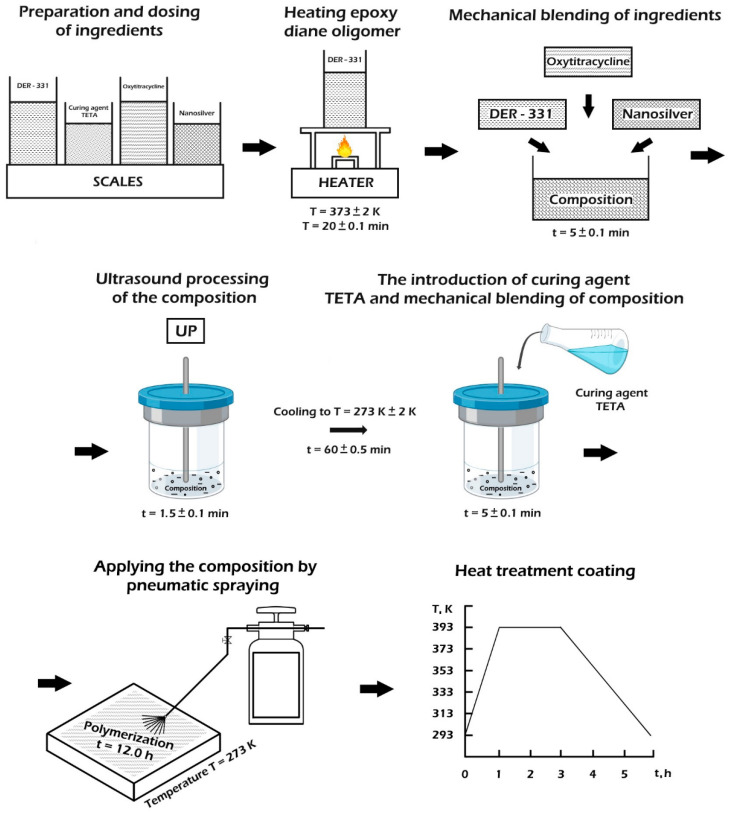
Scheme of temperature–time regimes of protective polymer coating formation, filled with nanosilver and oxytetracycline.

Figure 2 shows a schematic illustrating the process of composite formation. The sequence of synthesis of the epoxy composite includes the following steps:

- the preliminary preparation and dosing of ingredients
- the heating of epoxy resin DER-331 to a temperature of *T* = (373 ± 2) K and exposure for a specified time *τ* = (20 ± 0.1) min
- the mechanical mixing of ingredients during *τ* = (1.5 ± 0.1) min
- the ultrasonic treatment (UST) of the composition during *τ* = (1.5 ± 0.1) min;
- the cooling of the composition to room temperature *T* = (273 ± 2) K, during *τ* = (60 ± 5) min
- the adding the hardener TETA and mixing the composition for a time *τ* = (5 ± 0.1) min
- the application of the composition by mechanical or pneumatic spraying.
- the solidification of materials according to the experimentally established regime
- the formation of samples and their keeping during *t* = (12.0 ± 0.1) h at a temperature *T* = (293 ± 2) K
- heating at a rate of *υ* = 3 K/min to a temperature of *T* = (393 ± 2) K
- the keeping of the samples at a given temperature for a time *t* = (120 ± 5) min
- slow cooling to a temperature of *T* = (293 ± 2) K.

The change in the temperature regimes was not implemented as it would lead to a change in the results of the research unit filled with nanosilver and oxytetracycline.

For the stabilization of the structural processes in the matrix, the samples were kept for *t* = 24 h in the air at a temperature *T* = (293 ± 2) K. Then, they were coated with the epoxy resin DER-331 composite filled with nanosilver (at *q* = 0.5–1.5 wt%) and/or oxytetracycline (at *q* = 0.5–1.5 wt%). Pneumatic spraying was used for covering metal surfaces with composite materials.

### 2.2. Study of the Structure, Microrelief, and Chemical Composition of the Composite Surface

The ultimate tensile strength and flexural modulus were determined according to ASTM D 790-03. The sample parameters were length *l* = (120 ± 2) mm, width *b* = (15 ± 0.5) mm, and height *h* = (10 ± 0.5) mm. Studies of the structure and microrelief of surfaces were conducted using scanning electron microscopy (SEM) (the scanning electron microscope SEO-SEM Inspect S50-B; Sumy Electron Optics (SEO), Sumy, Ukraine) and X-ray energy dispersive spectroscopy (X-MaxN20: Oxford Instruments plc, Abingdon, Oxfordshire, UK).

### 2.3. Ecotoxicity Test Methods

The ecotoxicity of composite surfaces on metal test plates was determined using methods standard in eco-microbiology [44,45,46,47,48,49] on model systems with the typical intestinal microflora of humans and animals. The following microorganisms were used as test cultures in the model systems: probiotic strains of *Lactobacillus acidophilus* derived from the probiotic preparation Acidophilus Plus-Solgar (manufactured by Solgar, Inc., Leonia, NJ, USA); the *Bifidobacterium bifidum* strain, isolated from the probiotic preparation “Bifidumbacterinum-Biopharma” (manufacturer PJSC “BIOPHARMA”, Kyiv, Ukraine); and the *Escherichia coli* strain isolated from the aquatic environment in vitro. The identification of the microorganism was performed by studying the morphological, cultural, biochemical, and antigenic properties in the microbiological laboratory of Sumy State University (the certificate № RU-0213/19 from 15 March 2019).

### 2.4. Microbiological Methods for Studying Biofilm Formation

The standard method of Denisov, D. B. et al. [46] was used to determine the potential of bacterial cells to activate the initial stage of biofilm formation in the study of the adhesive properties of test cultures. Based on scientific data on the composition of marine microflora [18], as an inoculum for biofilms, 18 h test cultures of microorganisms were used. The first series of experiments aimed to a create two-component biofilm: heterophilic *Escherichia coli*, *Klebsiella pneumonia*, isolated from an aquatic environment. Based on scientific data on the composition of marine microflora [20], 18 h test cultures of microorganisms were used as an inoculum for biofilms. The first series of experiments aimed to create a two-component biofilm: heterophilic *Escherichia coli*, *Klebsiella pneumoniae*, isolated from an aquatic environment. During the second series of the experiment, a three-component biofilm was formed: test cultures of *Escherichia coli*, *Klebsiella pneumoniae* isolated from the aquatic environment and museum culture of *Aspergillus niger*. Each series of experiments was carried out five times with two-component and three-component biofilms. The reason for the design features of the second series of experiments is the fact that, in the natural marine environment, along with bacterial organisms, there are yeasts and microscopic fungi, which can also participate in biofilm formation and are microorganisms–bio-destructors.

The process of biofilm formation and the influence of the coating components of [36] metal test samples were evaluated by studying the reduction factor of the total volume of the formed biofilm. The results were considered for 2, 3, and 5 days. After cultivation, planktonic microflora cells were selected, and the metal test-samples were washed with a sterile buffer, which was then removed completely. For the colored formed biofilm, filtered 0.1% gentian violet (30 min) was added to the wells with metal test samples and decolorized with 80% ethanol solution (incubation temperature 25 °C). The optical density of the alcohol solution was measured using a spectrophotometer (*λ* = 595 nm, NanoDrop 2000®, Thermo Fischer Scientific, Waltham, MA, US). The reduction factor of the microbial biomass was calculated as the ratio of the optical density of the sample to the optical density of the control in percentage equivalent.

The biofilm-formation process on test samples was also studied using a scanning electron microscope: SEO-SEM Inspect S50-B with pre-spraying silver. Biofilm fixation was carried out using 2% glutaraldehyde solution. The dehydration of samples was performed in a series of ethanol concentrations (30%, 50%, 70%, 80%, 90%). The final dehydration of the samples was carried out in alcohol of maximum concentration (100%) for 15 min.

### 2.5. Statistical Methods

The statistical processing of the obtained results was performed using the software package for statistical data processing: Graph Pud Prism 8 (GraphPad Software 2365 Northside Dr. Suite 560 San Diego, CA, 92108, USA), with the definition of the Student’s criterion.

## 3. Results

### 3.1. Mathematical Planning of the Experiment

What is significant in the use of coatings to protect hulls is the ability to maintain adhesive and cohesive strength under the influence of environmental factors and to prevent the penetration of aggressive agents through the polymer to the base and the coatings’ ability to counteract fouling. Therefore, in the preliminary stages of research, the influence of fillers, namely nanosilver and oxytetracycline, on the physical–mechanical and thermophysical properties of the epoxy matrix relative to the content of each component was determined. By analyzing the obtained results, it can be asserted that composites containing the following ingredients (Table 1) are characterized by the following improved property indicators: 1. Oxytetracycline at *q* = 0.5 wt% (specified per 100 parts by weight of epoxy resin DER-331); 2. Oxytetracycline at *q* = 1.0 wt%; 3. Oxytetracycline at *q* = 1.5 wt%; 4. Nanosilver at *q* = 0.050 wt%; and 5. Nanosilver at *q* = 0.075 wt%; 6. Nanosilver at *q* = 0.100 wt%. It should be noted that we observed an improvement in the physical and mechanical parameters with the above-mentioned amounts of fillers in the epoxy oligomer. However, the thermophysical properties of the composite, which correspond to the values of the epoxy matrix, remain unchanged.

Additionally, two fillers in the complex (nanosilver and oxytetracycline) were used to improve the properties of the protective coatings, the optimal content of which was determined by the method of the mathematical planning of the experiment using the STATGRAPHICS^®^ Centurion XVI application package. Orthogonal central compositional planning was used to optimize the content of fillers of different nature to form the protective coating [50,51]. The content of the main and additional fillers was selected based on complex studies of the physical and mechanical properties of CM. Based on the study results of the cohesive strength of CM, Table 1 shows the main levels of change in the content of the components of NS and OT.

According to the experimental design scheme, nine experiments were performed (*N* = 9), each of which was repeated three times (*p* = 3) to rule out system errors (Table 2). In order to obtain an orthogonal planning matrix [50,51,52], the corrected level values, $x^{\prime }$, were entered and calculated using (1).

(1) $${x_{i}}^{\prime }={\left(x_{i}\right)}^{2}-\frac{\sum_{u-1}^{N}x_{iu}^{2}}{N}$$

The results of a complete factorial experiment with an extended planning matrix are shown in Table 3.

A mathematical model, *y* = *f* (*x*_1_, *x*_2_), was formed as a regression (see Equation (2)) with regression coefficients *b_i_* (2). The obtained coefficients of the regression equation are shown in Table 4.

(2) $$y=b_{0}^{}+b_{1}x_{1}+b_{2}x_{2}+b_{11}x_{1}^{2}+b_{22}x_{2}^{2}+b_{12}x_{1}x_{2};\;b_{i}=\frac{\sum_{u=1}^{N}x_{i}y_{i}}{\sum_{u-1}^{N}x_{iu}^{2}}$$

As a result of the analysis of the destructive stresses of CM, we observe the following regression Equation (3).

(3) $$y=82.21-0.60x_{1}-2.32x_{2}+3.53x_{1}^{2}-1.02x_{2}^{2}+3.25x_{1}x_{2}$$

For the statistical processing of the obtained experimental results, the reproducibility of experiments was tested by Cochran’s test (4).

(4) $$G=\frac{S_{u_{\max}}^{2}}{\sum_{u=1}^{N}S_{u}^{2}}\leq G_{\left(0.05;f_{1};f_{2}\right)},$$

where $S_{ui}^{2}$ is variance, which characterizes the scattering of experimental results on the *i*-th combination of factor levels for *m* = 3; *m* is the number of parallel experiments; and $S_{u_{\max}}^{2}$—the largest of the variances in the lines of the plan. 

The dispersion of adequacy was determined using Table 5 (5):

(5) $$S_{ui}^{2}=\frac{\sum_{i=1}^{m}{\left(y_{i}-\bar{y_{i}}\right)}^{2}}{m-1},$$

where $y_{im}$ is the value obtained in each parallel experiment, and $\bar{y_{i}}$ is the average value *y* obtained in parallel experiments.

Reproduction variances were determined using (6) and (7):

(6) $$\sigma^{2}\left\{y\right\}=\frac{\sum_{i=1}^{N=9}\sigma^{2}{\left\{y\right\}}_{i}}{N(m-1)},$$

where

(7) $$\sigma^{2}{\left\{y\right\}}_{i}=\sum_{i=1}^{m=3}{\left(y_{i}-\bar{y_{i}}\right)}^{2};\sigma^{2}\left\{y_{\mathrm{ave}}\right\}=\frac{a^{2}\{y\}}{N},\;\mathrm{or}\;S_{b_{0}}^{2}=\frac{S_{0}^{2}}{N}$$

The specific calculated values are:

(8) $$\sum_{i=1}^{N}S_{ui}^{2}=0.23;\sigma^{2}\left\{y\right\}=S_{0}^{2}=0.026.$$

From the viewpoint of the above-mentioned facts, the value of Cochran’s test at 5% equal significance can be represented by (9).

(9) $$G_{\mathrm{calc}}=\frac{S_{u_{\max}}^{2}}{\sum_{i=1}^{N}S_{ui}^{2}};G_{\mathrm{calc}}=\frac{0.04}{0.23}=0.174.$$

Thus, for a fixed probability of *α* = 0.05, verification of the results of experimental studies according to Cochran’s test [52] confirmed the reproducibility of the experiments. The dispersion characterizing the scattering of experimental results on the *i*-th combination of factor levels is $S_{u_{\max}}^{2}=0.04$. The tabular value of Cochran’s test is *G_calc_* = 0.174. The tabular value of Cochran’s test is *G_table_* = 0.478. Comparing the obtained values, we fulfil the following conditions:

(10) $$G_{\mathrm{calc}}=0.174\leq G_{tabl}{}_{e}=0.478$$

The next step was to analyze the results according to the experimental design, determining the significance of the coefficients of the regression equation (Table 6).

Subsequently, the variances of the regression coefficients were determined (Table 7) according to (11).

(11) $$S_{b_{i}}^{2}=\frac{S_{0}^{2}}{\sum_{u-1}^{N}x_{iu}^{2}}$$

The value of the regression coefficients was determined by the Student’s *t*-test [52,53]. Collectively, both the tabular (*t_m_*) and calculated (*t_p_*) *t*-tests were determined (Table 7).

Depending on the degrees of freedom: *f* = *N* (*n* – 1) = 9 (3 – 1) = 18, the tabular value of the *t*-test was determined, which is *t_T_* = 2.1.

The calculated values of the *t*-test (*t_p_*) and the significance of the coefficients were determined: *t*_0*p*_, *t*_1*p*_, *t*_2*p*_, *t*_11*p*_, *t*_22*p*_, *t*_12*p*_ > *t_T_*.

This is represented in (12):

(12) $$t_{ip}=\frac{\left|b_{i}\right|}{S_{b_{i}}^{}}$$

Since the calculated values of the Student’s *t*-tests *t*_0*p*_, *t*_1*p*_, *t*_2*p*_, *t*_12*p*_, *t*_11*p*_, *t*_22*p*_ are higher than *t_T_*, we consider that all coefficients of the regression equation *b*_0_, *b*_1_, *b*_2_, *b*_12_, *b*_11_, *b*_22_ are significant. Therefore, the regression equation has its original form (13).

(13) $$y=82.21-0.60x_{1}-2.32x_{2}+3.53x_{1}^{2}-1.02x_{2}^{2}+3.25x_{1}x_{2}$$

According to Fisher’s test, the adequacy of the obtained model was checked (14) [51].

(14) $$F_{p}=\frac{S_{u_{\max}}^{2}}{S_{y}^{2}}\leq F_{\left(0.05;f_{a\partial };f_{y}\right)},$$

where $S_{u_{\max}}^{2}=0.04$ is the calculated value of the dispersion adequacy (Table 5) (15).

(15) $$S_{y}^{2}=\frac{\sum_{i=1}^{N}S_{ui}^{2}}{N},$$

where *S_y_*^2^ = 0.026 is the variance of reproducibility.

Therefore, *F_p_* = 1.565. $F_{\left(0.05;f_{a\partial };f_{u}\right)}$ The Fisher’s test tabulated value for the “5%” reliability level (*f*_1_ = *N* − (*k* + 1) = 9 − (6 + 1) = 2, *f*_2_ = *N* (*n* − 1) = 9 (3 − 1) = 18). This is represented by *F*_(*t*)_ = 3.55 [52,53].

Comparing the calculated value of Fisher’s test with the tabular, we see the fulfilment of condition (13). It can be assumed that the equation reliably describes the composition. As a rule, the process of the interpretation of the resulting mathematical model is not limited to determining the influence of factors. A simple comparison of the absolute value of linear coefficients does not determine the relative degree of influence of factors, since there are also quadratic terms and pairwise interactions. In a detailed analysis of the obtained adequate model, it should be considered that, for the quadratic model, the degree of influence of the factor on the change of the initial value is not constant.

Consider the following dependencies that connect the normalized and natural values of the variables [51].

(16) $$x_{i}=\frac{q_{i}-q_{i0}}{\Delta q_{i}}$$

where *q_i_* represents the value of the i-th experiment factor, *q_i0_* represents the value of zero level, and Δ*q_i_* represents the interval of variation [43].

According to (16), replacing these values in the regression equation, and then converting them, we obtained a regression equation with a natural value of variable parameters (17).

(17) $$R_{bm}=114.8-49.0q_{1}^{}-108.7q_{2}^{}+14.1q_{1}^{2}-1626.7q_{2}^{2}+260.0q_{1}q_{2}$$

**Figure 3:**
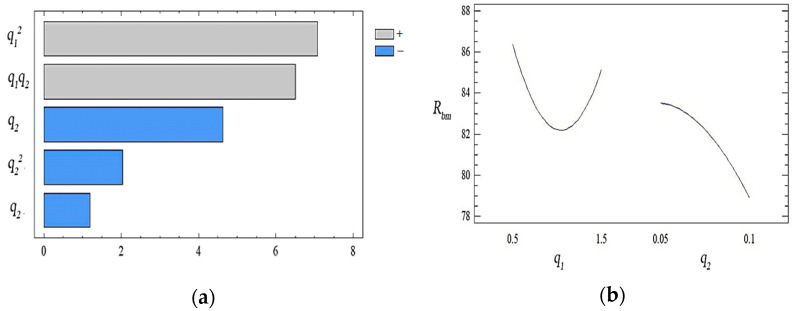
(**a**) The set of Pareto efficient solutions; (**b**) main effects of *R_bm_*.

**Figure 4:**
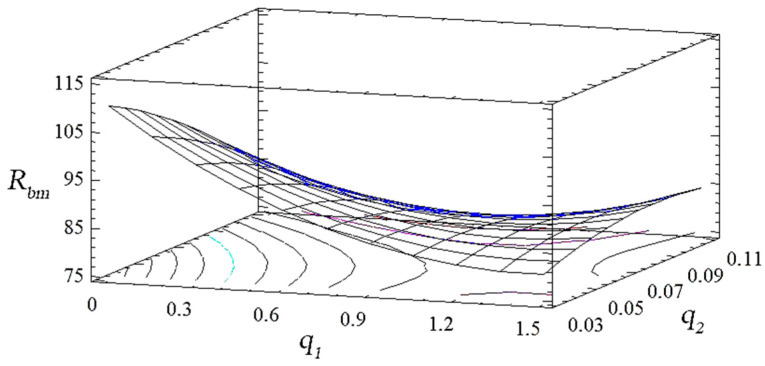
Derived response surface of *R_bm_* = *f* (*q*_1_, *q*_2_).

**Figure 5:**
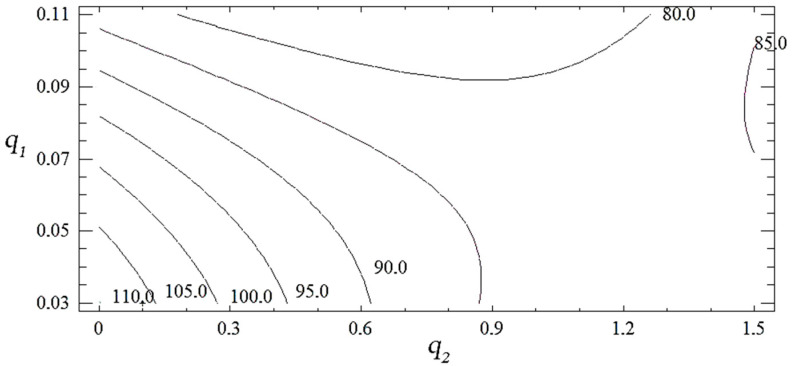
Contours of the calculated response surface *R_bm_* = *f* (*q*_1_, *q*_2_).

With natural values, this equation allows you to predict only the value of the original value for any point that is within the range of factors. Despite the above, it can be used to plot the dependence of the initial value (yield strength during bending) on any factor (or two factors). The geometric interpretation of the obtained values is shown in the form of the response surface (Figure 3, Figure 4 and Figure 5). During the experiment, the importance of both factors was established. It can be emphasized that the value of the ultimate strength during bending has the greatest influence on the quadratic dependence of the first factor (oxytetracycline) and the product of two factors (according to the Pareto map). When the calculated response surface was analyzed, it was found that the optimal bending strength is characterized by an epoxy composite with the following content of fillers: oxytetracycline—0.5 of the mass fraction and nanosilver—0.050 of the mass fraction (*R_bm_* = 92.4 Mpa).

According to the previous calculations, the components of the composition were optimized according to the modulus of elasticity in bending. According to Table 1 and Table 2, the experiment’s planning scheme and levels of variables in conditional and natural scales were chosen. When the results of the modulus of elasticity of the composite material were investigated, regression equation coefficients were obtained, as shown in Table 8. 

As a result of the regression, the equation is as follows (18).

(18) $$y=2.97-0.02x_{1}-0.05x_{2}+0.15x_{1}^{2}-0.25x_{2}^{2}+0.10x_{1}x_{2}$$

According to the Cochran test (4), the reproducibility of experiments was checked for the statistical processing of the obtained experimental results.

The values of the variances determined by (6) and (7) are shown in Table 9.

The calculated value of the Cochran test was found by (8) and then compared with tabular data. The verification of the results of the experiment by the Cochran test for a fixed probability *ψ* = 0.05 confirmed the reproducibility of the experiments. The dispersion characterizing the scattering of the experimental results on the *i*-th combination of factor levels is $S_{u_{\max}}^{2}=0.020$.

The calculated value of the Cochran test is *G*_*po*3*p*_ = 0.295. Accordingly, the tabular value of the Cochran test is *G_table_* = 0.478. That is, the condition is fulfilled, so *G*_*po*3*p*_ = 0.295 ≤ *G_table_* = 0.48.

Next, the value of the coefficients of the regression equation was determined by analyzing the results according to the experimental design (Table 10). The tabular value of the Student’s test is *t_T_* = 2.1. Subsequently, the dispersion of the regression coefficients (9) and the calculated values of the Student’s *t*-test (10) were determined (Table 11).

According to the Student’s *t*-tests, the values of *t*_0*p*_, *t*_11*p*_, *t*_22*p*_, *t*_12*p*_ calculated above are greater than *t_T_*, so the coefficients of the regression equation *b*_0_, *b*_11_, *b*_22_, *b*_12_ are significant. Since the values of *t*_1*p*_ and *t*_2*p*_ of the Student’s *t* test are less than *t_T_*, we can consider the indices of the coefficients of the regression equation *b*_1_, *b*_2_ to be insignificant. After discarding all of the insignificant coefficients, the regression equation is as follows (19).

(19) $$y=2.97+0.15x_{1}^{2}-0.25x_{2}^{2}+0.10x_{1}x_{2}$$

Using Fisher’s test, we tested the adequacy of the obtained model (13), (14). In the process of work, its tabular and calculated values were determined. According to Fisher’s test, the tabular value at the 5% significance level is *F*(*t*) = 2.93 and is higher than the calculated value (*F_p_* = 2.657) under which condition (12) is satisfied. As mentioned above, the regression equation adequately describes the composition. When converting the elements of the regression equation according to (14), we obtained the following equation using the natural value of the variable parameters (20).

(20) $$E=2.1-1.83333q_{1}+50.0q_{2}+0.6q_{1}^{2}-400.0q_{2}^{2}+8.0q_{1}q_{2}$$

**Figure 6:**
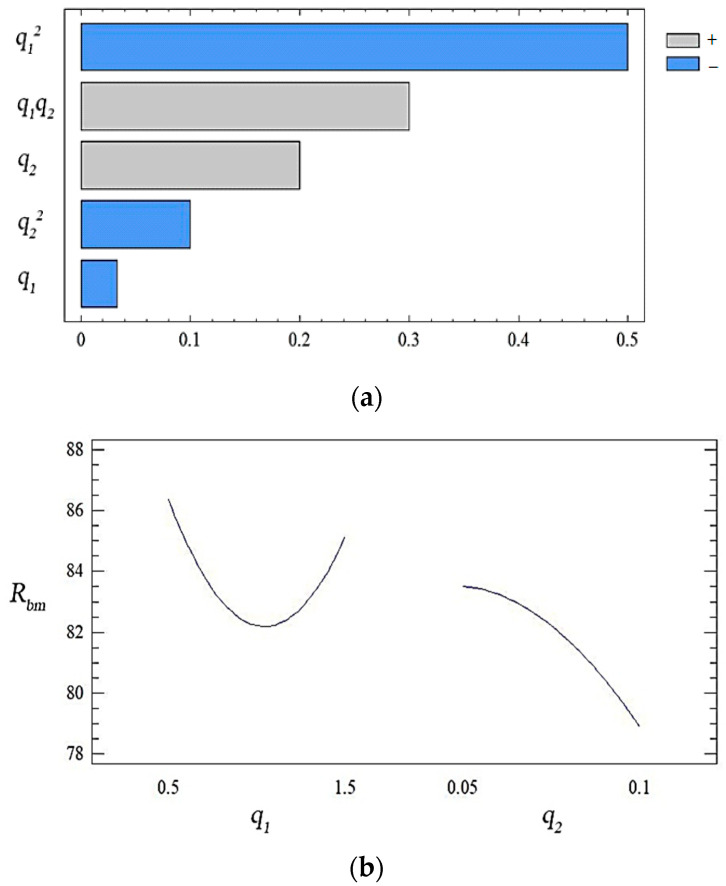
(**a**) The set of Pareto efficient solutions; (**b**) main effects of *E*.

During experimental research, the value of both factors was determined. We emphasize that according to the analysis of the Pareto map, it can be argued that the effect of nanosilver on these indicators of the modulus of elasticity is more pronounced than the influence of oxytetracycline (Figure 6).

**Figure 7:**
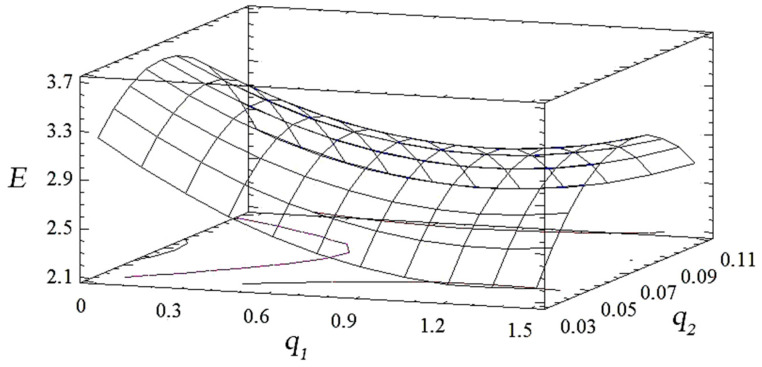
Derived response surface of *E* = *f* (*q*_1_, *q*_2_).

**Figure 8:**
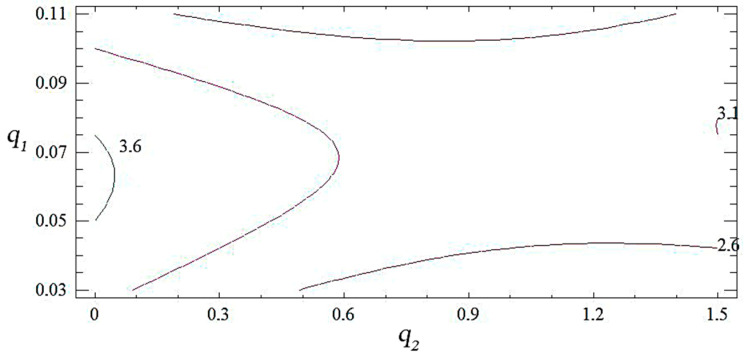
Contours of the calculated response surface *E* = *f* (*q*_1_, *q*_2_).

Analyzing the combinations of experimental factors using the calculated response surface (Figure 6, Figure 7 and Figure 8), we observed the maximum values of the modulus of elasticity in bending for the content of fillers: nanosilver—0.075 wt%, oxytetracycline—1.5 wt% (*E* = 3.3 GPa). The change in the ratio of the content of the filler deteriorates the modulus of elasticity during bending. Therefore, on the basis of complex research and the mathematical planning of the experiment, variants of coatings were established, which were studied for ecotoxicity and biofilm formation (Table 12).

### 3.2. The Structure and Microrelief of Surfaces

**Figure 9:**
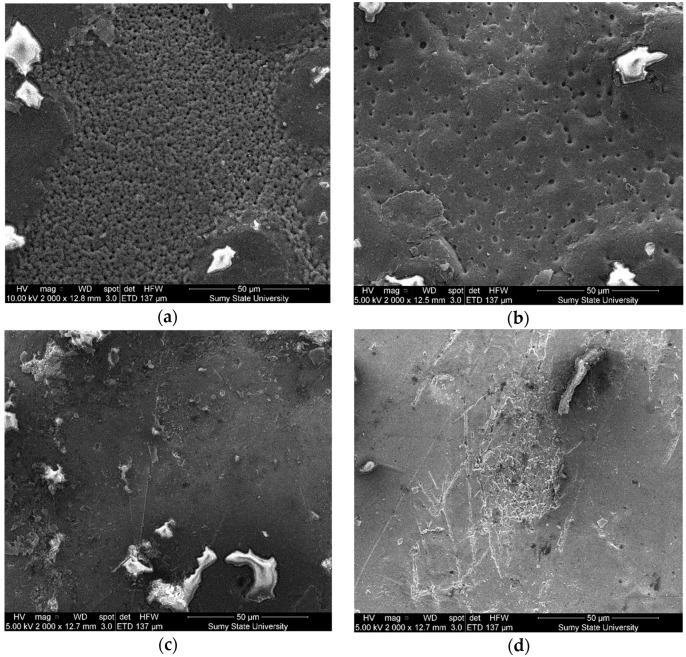
SEM images of the morphology of the surfaces of the composite material: (**a**) epoxy matrix DER-331; (**b**) DER-331 with oxytetracycline at *q* = 1.5 wt% and nanosilver at *q* = 0.075 wt%; (**c**) DER-331 with oxytetracycline at *q* = 1.5 wt%; (**d**) DER-331 with nanosilver at *q* = 0.075 wt%.

The study results of the structure and microrelief of surfaces (scanning electron microscopy) of composite materials are shown in Figure 9. The analysis of the structure of the epoxy matrix (Figure 9a) allows us to assert the homogeneity of the polymer. The analysis of the composite surface filled with oxytetracycline particles (*q* = 1.5 wt%) and nanosilver (*q* = 0.075 wt%) allowed us to identify significant differences in surface morphology (Figure 9b). The nano-porous structure of coatings with dimensions ranging from 0.1−1.0 nm has been established. It was believed that the formation of homogeneous pores was associated with the uniform distribution of silver particles in the volume of the polymer matrix, which is provided at the stage of composition formation due to complex formation, accompanied by intermolecular contacts between binder and metal ions. During polymerization, a nano-heterogeneous structure of coatings is formed in the presence of silver particles and oxytetracycline. It was considered that the porous, nano-heterogeneous structure of the layers would increase the release rate of silver ions from the polymer surface. In this case, silver nanoparticles can contact the cell wall of bacteria and are able to penetrate into the cytoplasm. It provides changes in the cell membrane and inner structures and, subsequently, cell death. This, in turn, prevents the growth of microorganisms. Additionally, it was found that there are no pronounced directional chipping lines for the studied materials, which indicates insignificant residual stresses in the composite. This fact further confirms the high physical and mechanical properties of composite materials (Table 3). Coatings where antibacterial additives were used separately (Figure 9c,d) are characterized by a slightly different surface relief with a small agglomeration of the micro- and nano-dispersed component of biocides.

### 3.3. The Chemical Composition of the Composite Surface

**Figure 10:**
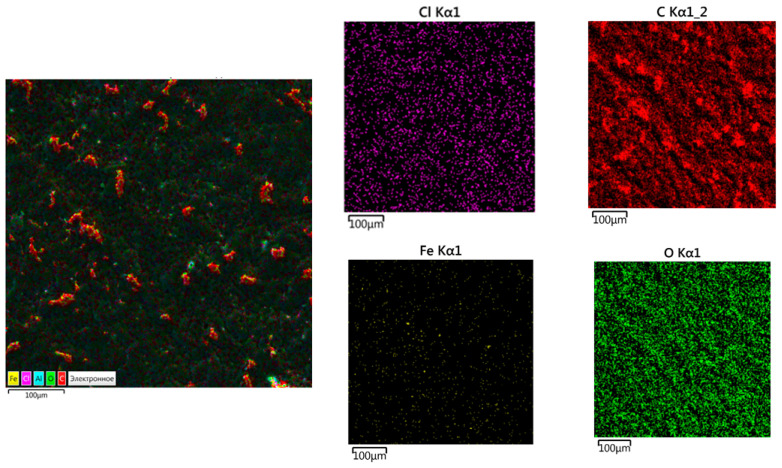
Analysis of the test sample using X-ray energy dispersive spectroscopy.

The study results of the sample (oxytetracycline at *q* = 1.5 wt% and nanosilver at *q* = 0.075 wt%) using X-ray energy dispersive spectroscopy are shown in Figure 10. When the chemical composition of the composite surface was analyzed, the following elements were found: Cl, Fe, C, and O. It was believed that the well-defined areas of carbon (C) detected by X-ray energy dispersion spectroscopy were part of the epoxy matrix. Given that carbon is one of the components of the polymeric material, the weight fraction C should occupy the maximum surface area. Concerning oxygen (O), it can be attributed both to the epoxy matrix and to additive–biocides (oxytetracycline or nanosilver). The presence of a metallic component (Fe) was also observed on the surface of the protective coating. The assumption was made that Fe can be attributed to nanosilver impurities. At the same time, chlorine (Cl) is one of the components of oxytetracycline [52,53,54].

### 3.4. Ecotoxicity Analysis of Polymer Coatings with Biocidal Fillers

The design of the experiment to study the ecotoxicity of the studied composite surfaces and the process of biofilm formation considered the fact that nanosilver and oxytetracycline have antibacterial properties. Simultaneously, the toxic effect of silver nanoparticles on living organisms is known [34,55]. In biotic doses, nanosilver, on the other hand, has positive effects; it can stimulate metabolism and weight gain [56], increase the number of bifidobacteria in the microflora of the gastrointestinal tract [57], and improve the antioxidant status of the body [14]. The analysis of the studied sample using the X-ray energy dispersion spectroscopy method is shown in Figure 10.

Given all the above, we conducted an ecotoxicity in vitro analysis of samples using laboratory and analytical studies generally accepted in microbiology [15] in model systems with typical intestinal microflora (probiotic strains of *Lactobacillus acidophilus*, *Bifidobacterium bifidia*, and *Escherichia coli* isolated from the aquatic environment). The results of the in vitro tests used to assess the safety of nanomaterials in these model systems are shown in Table 13. 

When comparing the results of in vitro tests to assess the safety of test specimens coated with nanosilver and/or oxytetracycline, the time of the physiological stages of propagation of pure test cultures in a liquid medium with an uncoated metal test plate was considered. We found that a metal test plate with a coating containing oxytetracycline (*q* = 1.5 wt%) and nanosilver (*q* = 0.075 wt%) inhibits probiotic strains of *L. acidophilus*, *E. coli*, as there is a significant reduction in CFU/mL test strains in media with the tested nanomaterial and in media without it by at least one logarithmic order in the in vitro model. A toxic effect on this probiotic test culture was recorded for this sample. For all other samples of test plates with nanosilver and/or oxytetracycline of different concentrations, no toxic effect was recorded on the model system of typical intestinal microflora.

The fact that test cultures of the model system of typical intestinal microflora in the presence of tested coated metal plates have a significantly lower (*p* ≤ 0.05) a number of bacterial cells in 1 mL of nutrient medium compared to the controlled growth of test cultures in the thioglycolic medium for 72 h of cultivation is noteworthy. It can indirectly indicate the leaching of the coating fillers (oxytetracycline or nanosilver) of the metal test plates and the bacteriostatic effect of the formed medium on the test cultures.

To confirm this assumption, we studied the adhesive properties of test cultures of the typical intestinal microflora model system’s working suspension before co-cultivation with the tested coated metal test plates 72 h after cultivation with test samples.

By analyzing the study results of the adhesive properties of the test cultures used in in vitro tests for the ecotoxicity of the fillers of the coating of metal plates (*Lactobacillus acidophilus*, *Bifidobacterium bifidum*, *and Escherichia coli*), it was found that the original strains of these test cultures had high adhesive power (IAM ≥ 7 bact. Cells/erythrocyte). After 72 h of cultivation with samples of tested metal plates, the level of adhesion significantly (*p* ≤ 0.05) decreased. Thus, *Lactobacillus acidophilus*, which showed a high level of adhesion, had a medium degree of adhesion after 72 h (IAM 3.5 bact.cells/erythrocyte); *Bifidobacterium bifidum* had a low degree of adhesion (IAM 1.95 bact.cells/erythrocyte); and *Escherichia coli* test culture, which had the highest level of adhesion at the beginning of the experiment (IAM 8.3 bact.cells/erythrocyte), reduced the adhesive potential to a low level (IAM 2.0 bact.cells/erythrocyte). The obtained data indicate that, after the prolonged contact of the test culture with the coating of metal plates or due to the leaching of the components of this coating, planktonic microflora change their adhesive properties to reduce the adhesive potential. These results indicate the suppression of the initial stage of development of biofilms, namely the attachment of bacteria to the biotic or abiotic surface, inhibiting the transition to a biofilm lifestyle.

### 3.5. Analysis of Biofilm Formation on Polymer Coatings with Biocidal Fillers

**Figure 11:**
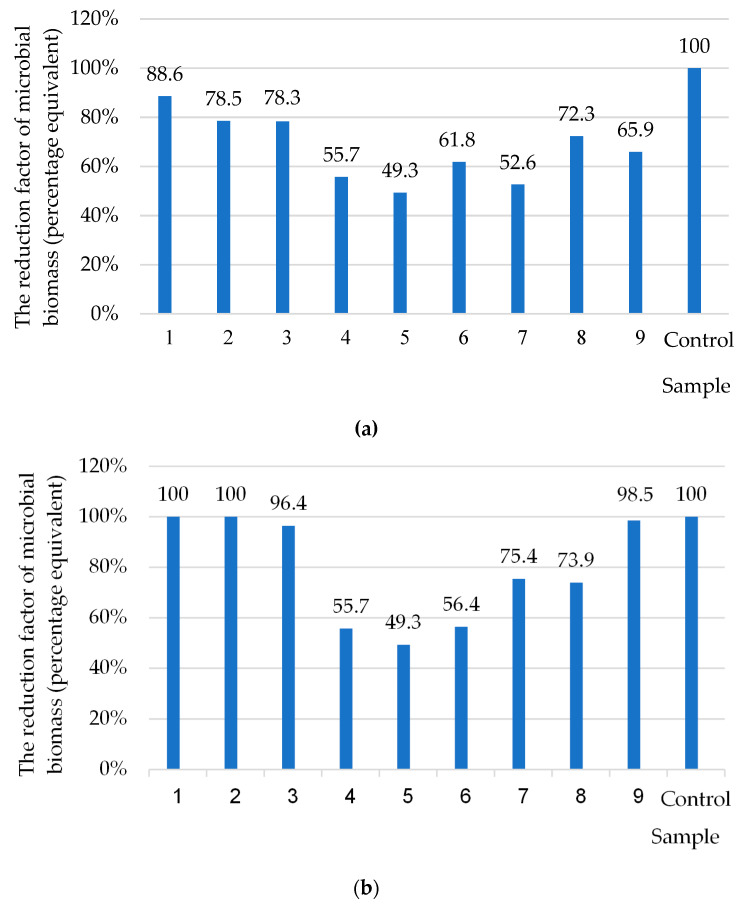
The results of the influence of the fillers of the coating of metal samples on mixed biofilm formation: (**a**) two-component biofilm (120 h): *Escherichia coli* and *Klebsiella pneumoniae*, isolated from the aquatic environment; (**b**) three-component biofilm: *Escherichia coli* and *Klebsiella pneumoniae*, isolated from the aquatic environment, and *Aspergillus niger* (museum culture). Fillers (oxytetracycline—OT; Nanosilver—NS) in the coating: Sample 1—OT *q* = 0.5 wt%; Sample 2—OT *q* = 1.0 wt%; Sample 3—OT *q* = 1.5 wt%; Sample 4—NS *q* = 0.050 wt%; Sample 5—NS *q* = 0.075 wt%; Sample 6—NS *q* = 0.100 wt%; Sample 7—OT *q* = 0.5 wt% and NS *q* = 0.075 wt%; Sample 8—OT *q* = 0.5 wt% and NS *q* = 0.050 wt%; Sample 9—OT *q* = 1.5 wt% and NS *q* = 0.075 wt%; the control steel sample is without coating (Steel Grade-Fe360-C).

One of the most critical factors in implementing the adhesion process is the roughness of the surface of the substrate on which the biofilm is formed. Therefore, the fundamental aspect of the study of biofilm formation is the study of fundamentally different biological mechanisms such as adhesion and colonization. By analyzing the results of biofilms on the surface of coated metal plates by the coefficient of the reduction of the total volume of biofilms, which included nanosilver and/or oxytetracycline, it was found that the fillers of the coating have different effects on the level of biofilm after 120 h of the experiment (Figure 11a,b).

**Figure 12:**
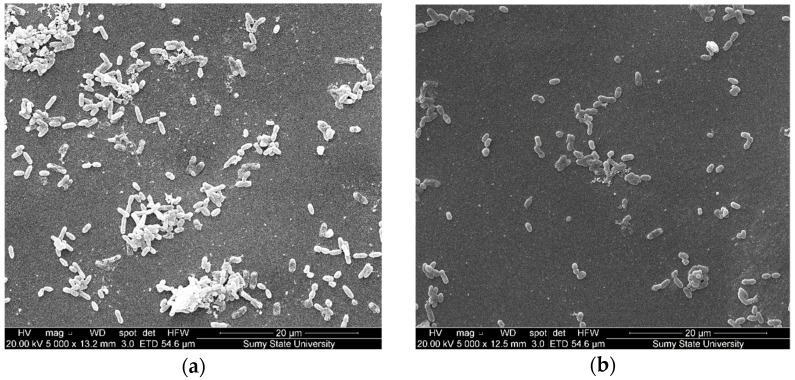
Results of scanning microscopy of samples (120 h of formation of two-component bacterial biofilm on the test samples surface): the metal sample with a coating containing nanosilver at *q* = 0.050 wt%: (**a**) nanosilver at *q* = 0.075 wt%; (**b**) (dispersion 10–100 nm); the first series of the experiment.

The data obtained (Figure 11a) show that the fillers of the coating reduce the level of adhesion of the test cultures (Table 13) and inhibit the formation of biofilms of bacterial etiology after 120 h of the experiment. A significant decrease in the total volume of the formed biofilm by 120 h of the experiment was revealed (Figure 11a). The results obtained by scanning electron microscopy confirmed that there was a decrease in biomass biofilm on a metal test sample with nanosilver at *q* = 0.050 wt% (Figure 12a) and with nanosilver at *q* = 0.075 wt% (Figure 12b) after 120 h of the experiment.

**Figure 13:**
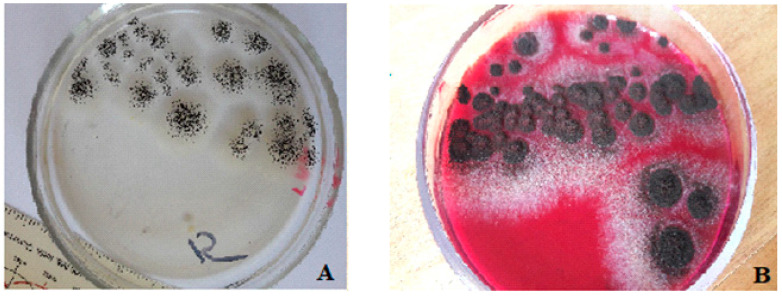
Control seeding of the biofilm material (72 h) from the sample surface with the oxytetracycline filler at *q* = 1.0 wt% on the following: (**A**) Sabouraud agar: *Aspergillus niger* colonies; (**B**) Endo medium: *Aspergillus niger* colonies; without *Escherichia coli* and *Klebsiella pneumoniae* colonies.

**Figure 14:**
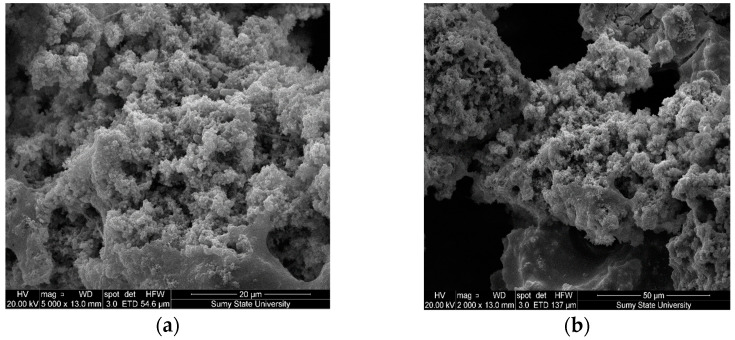
Results of scanning microscopy of samples (72 h of formation of a three-component bacterial-fungal biofilm): (**a**) DER-331 with oxytetracycline at *q* = 0.5 wt% and nanosilver at *q* = 0.075 wt%; (**b**) DER-331 with oxytetracycline at *q* = 1.5 wt%.

The analysis of the dynamics of the formation of a three-component bacterial–fungal biofilm (the second series of experiments) showed that, in the early incubation period (72 h) and on the surface of samples with oxytetracycline (sample 1, 2, 3, Figure 11b), biofilm was formed only by *Aspergillus niger.* The bacterial cells of *Escherichia coli* and *Klebsiella pneumoniae* in the biofilm were not detected both during the control seeding of the biofilm material on the differential diagnostic medium Endo (Figure 13) and in the study of all coatings using SEM (Figure 14).

The obtained result can indicate that the long-term incubation of these test metal plates containing the antibiotic filler stimulates the growth of fungal microbiota, namely the fungus-biodestructor—*Aspergillus niger*. It should be noted that the biofilms did not contain *Aspergillus niger* mycelium, and they were only found within spores of this fungus (Figure 14). On the other hand, such changes in the microbial composition during biofilm formation may be due to the fact that oxytetracycline, which is part of the polymer coating, exhibits a bactericidal effect upon prolonged contact with a bacterial cell. That is, there is a violation of *Quorum sensing*. Under natural conditions, multi-species biofilms are the predominant form of the existence of microbial “communities” (*Quorum sensing*) in the long term. The forming and components of the biofilm are influenced by various factors. Adding nanoparticles to coatings changes the physicochemical properties of active substances, which in turn changes the behavior of microorganisms in forming these “communities”. The direction of change is difficult to predict, as nanoparticles and nanomaterials are characterized by properties such as emergence.

**Figure 15:**
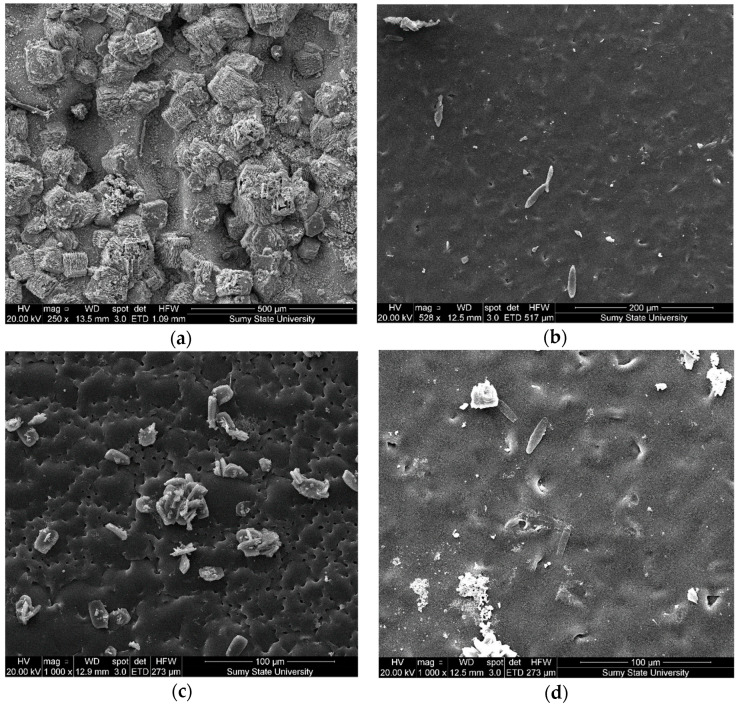
Results of scanning microscopy of samples for the formation of a two-component bacterial biofilm over the course of 14 days: (**a**) control steel sample (without coating; Steel Grade-Fe360-C); (**b**) DER-331 with nanosilver at *q* = 0.075 wt% (dispersion is 10–100 nm); (**c**) DER-331 with oxytetracycline at *q* = 1.5 wt% and nanosilver at *q* = 0.050 wt%; (**d**) DER-331 with nanosilver at *q* = 0.075 wt% (dispersion is 10–100 nm).

In connection with the above and previous results, for the purposes of microbiological and analytical assessments of the long-term impact of coating components of test samples on biofilms, we conducted an additional series of experiments with the formation of biofilms on test samples over 14 days. The experiment results with the two-component biofilm indicate that, when increasing incubation time to 14 days, there is a decrease in the number of biofilm-forming cells *Escherichia coli* and *Klebsiella pneumonia* (compared to 120 h of the experiment) on metal test plates. Microorganisms were found in the typical vegetative form without signs of active division (Figure 15a) and without an exocellular matrix, which is confirmed by the results of the SEM.

The change in the bio-filming process of *Escherichia coli* and *Klebsiella pneumoniae* test cultures in model systems after increasing the incubation time to 14 days may be due to the influence of a coating of nanoparticles on the activity of microorganisms. With prolonged exposure of bacterial cells in the coating of metal test samples, nanoparticles of fillers can penetrate into the cytosol of cells; change the functioning of membrane structures, nucleic acids, and proteins; and promote reactive oxygen species (ROS) generation. Changing the functioning of cell biostructures leads to the inhibition of the last stage of the formation of mature biofilms with the construction of a massive exocellular matrix. Additionally, research reports have demonstrated that the Ag+ ions firmly connect with the thiol (-SH) links of crucial compounds and disable them [24]. It is known that the exocellular matrix performs a protective function, actively participates in the formation of mixed biofilms, and prevents the diffusion of bactericidal agents and the action of damaging physical environmental factors. The absence of a full-fledged exocellular matrix (Figure 15b–d) in the formation of two-component biofilms confirms that coatings with fillers reduce the adhesive properties of test cultures, confirmed by in vitro tests. The structural and physicochemical features of surfaces affect the speed and quality of the subsequent stages of biofilm formation, including the construction of a mature biofilm. It should be noted that, on the surface of metal samples coated with a composite material based on epoxy oligomer DER-331 with additive–biocides, there is no pronounced exocellular matrix (nanosilver at *q* = 0.075 wt% (Figure 15b); with oxytetracycline at *q* = 1.5 wt% and nanosilver at *q* = 0.050 wt% (Figure 15c); nanosilver at *q* = 0.075 wt% (Figure 15d).

**Figure 16:**
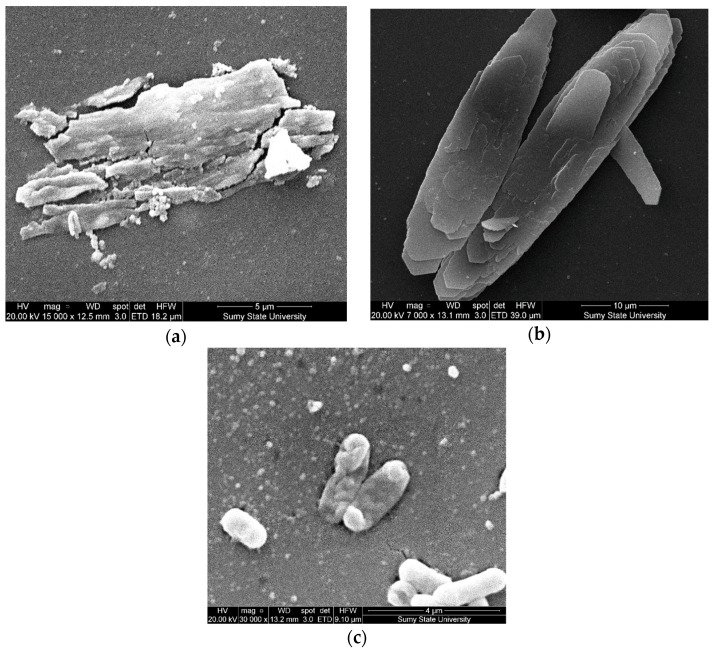
Results of scanning microscopy of samples (14 days of the two-component bacterial biofilm formation on the surface of metal test samples), the shape of test culture cells (destruction): (**a**) DER-331with nanosilver on the content *q* = 0.050 wt%; (**b**) DER-331with nanosilver at *q* = 0.075 wt%; (**c**) DER-331 with oxytetracycline at *q* = 0.5 wt% and with nanosilver at *q* = 0.050 wt%.

The bacterial cells of biofilm-forming test cultures include destroyed cells (Figure 16a–d). From our perspective, this confirms the fact that these biofilm-forming bacteria cells are destroyed due to prolonged contact with the above-mentioned composite materials. Eventually, it leads to the suppression of the formation and maturation of the biofilm and, as a result, to the inhibition of the process of the biofouling of surfaces. In addition, these results may be related to the fact that silver ions have a high affinity for electron-donating groups that are widely found in cell membranes or proteins, such as sulfhydryl, carbonyl, and phosphate groups. They can also bind to protein thiol groups, change their three-dimensional structure, and thus block active binding sites. The particular structure and multiple ways of contact with the bacterial cell membrane give them a unique way of counteracting the growth of bacteria [25].

Finally, in the experimental part, it can be stated that the demonstration of the SEM results makes it possible to see the result of the influence of the polymer-coating components and to understand the reason for the absence of an active biofilm formation process in each case. Figure 12 shows the same object magnification, allowing us to compare the degree of biofilm formation. The magnified images in Figure 14 demonstrate the components of the fungal test culture and demonstrate the revealed effect of the coating nanocomponents on the bacterial test culture. The absence of a full exocellular matrix (Figure 15b–d) during the formation of two-component biofilms confirms that coatings with fillers reduce the adhesion properties of test cultures, confirmed by in vitro tests. The structural and physicochemical properties of the coatings affect the rate and quality of the subsequent stages of biofilm formation, including the construction of the mature biofilm.

## 4. Conclusions

Three test cultures of probiotic bacterial strains were used to study the ecotoxicity of the tested samples (reported in the manuscript of the article): the probiotic strains of *Lacto-bacillus acidophilus* derived from the probiotic preparation Acidophilus Plus-Solgar (manufacturer Solgar, Inc., USA); the *Bifidobacterium bifidum* strain, isolated from the probiotic preparation “Bifidobacterium-Biopharma” (manufacturer PJSC “BIOPHARMA”, Ukraine); and the *Escherichia coli* strain isolated from the aquatic environment in vitro. The microorganism was identified by studying morphological, cultural, biochemical, and antigenic properties in the microbiological laboratory of Sumy State University (certificate No. RU-0213/19 dated 15 March 2019).

Three test cultures were also used in the study of biofilm formation as the main process of biodegradation (reported in the manuscript of the article). Each series of experiments was performed five times with two- and three-component biofilms.

The first series of experiments aimed to create a two-component biofilm: heterophilic *Escherichia coli*, *Klebsiella pneumonia*, isolated from an aqueous environment. Based on scientific data about the composition of marine microflora [18], 18 h test cultures of microorganisms were used as the inoculum for biofilms. The first series of experiments aimed to create a two-component biofilm: heterophilic *Escherichia coli*, *Klebsiella pneumoniae*, isolated from an aquatic environment.

The second series of experiments produced a ternary biofilm: test cultures of *Escherichia coli*, *Klebsiella pneumoniae*, isolated from an aqueous environment and a museum culture of *Aspergillus niger*. The reason for the design features of the second series of experiments is that in the natural marine environment, along with bacterial organisms; there are yeasts and microscopic fungi that can also participate in biofilm formation and are classified as “microorganism-biodestructors”.

The content of different additive–biocides, which differ by physicochemical properties and the dispersion in the epoxy matrix DER-331, was optimized by the method of the mathematical planning of the experiment to obtain functional coatings. The optimal content of basic (oxytetracycline) and additional (nanosilver) fillers for forming composites with high-cohesion characteristics has been established. It was proved that the introduction of oxytetracycline at *q* = 1.5 wt% and nanosilver at *q* = 0.075 wt% per 100 parts by weight oligomer DER-331 and 10 wt% of the TETA hardener provided the formation of a material with the following properties: flexural strength—*R_bm_* = 87.7 MPa, flexural modulus—*E* = 3.3 GPa. The increase in mechanical characteristics is associated with the change of the polymer structure because of the compaction of the spatial network of the polymer, which ensures the moderate mobility of the segments and side groups of the composite macromolecules.

The analysis of the results of the in vitro testing of surfaces formed using a composite material based on epoxy oligomer DER-331 with additive–biocides (nanosilver and/or oxytetracycline) showed toxic effects on *Escherichia coli* and probiotic strain *Lactobacillus acidophilus* only for samples with oxytetracycline at *q* = 1.5 wt% and with nanosilver at *q* = 0.075 wt%. All other samples of metal test plates with different concentrations of nanosilver and/or oxytetracycline in the composite coating do not have a toxic effect on the typical intestinal microflora.

After prolonged contact with the coatings of the metal test samples, the planktonic microflora change their adhesive properties. The decreased adhesive potential inhibits the initial stage of biofilm development (the attachment of bacteria to the biotic or abiotic surface) and represses the transition to a biofilm lifestyle. The dynamics of the processes in the initial stages of biofilm formation indicate that silver nanoparticles (in concentrations that inhibit the growth and reproduction of microbiota), as part of composite coatings based on epoxy oligomer DER-331, can be a factor of decreasing in biofouling. In perspective, these properties of the new composite material based on epoxy oligomer DER-331 allow us to recommend the designed coatings to protect the bodies of water transport vessels from fouling and destruction by microorganisms.

## 5. Perspectives for Future Research

Seawater is a specific habitat for microorganisms associated with the characteristic salt composition of water, low temperatures, high pressure, low concentrations of organic matter, and more. Additionally, the waters of some seas contain significant concentrations of hydrogen sulfide. Thus, in the Black Sea, there is a hydrogen sulfide zone, in which the amount of hydrogen sulfide increases with depth. Hydrogen sulfide in water is formed solely by the activity of microorganisms because of two processes—ammonification (decomposition) of proteins by anaerobic putrefactive microorganisms, and desulfation—i.e., the reduction of salts of sulfuric and sulfurous acids with the formation of hydrogen sulfide in the presence of organic matter. Both processes are equally active. 

Toxic effects on the environment and marine life are currently manifested by an accelerated reduction in ocean pH, mainly due to the excessive burning of fossil fuels. More than a third of excess CO_2_ is emitted into the atmosphere and then dissolved in seawater to form H_2_CO_3_, increasing its acidity. Its slightly alkaline nature is increasingly approaching neutral pH values, with a number of serious consequences for marine organisms [44].

All these factors have a long-term impact, which causes the biological fouling of ships/structures, significantly impairs the efficiency of use of these facilities, and increases the economic costs associated with their maintenance and operation. Based on the above, the prospects for further research are to study the structural changes of nanomaterial-based coatings in modeling systems for biofilm formation in the long term (3–4 months), taking into account the chemical composition of seawater and the inclusion of algae in the model test system.

The results of the microbiological studies of the biofilm formation could not be compared with data from other studies because the present experiment used information on protective polymer coatings with a combination of nanosilver and oxytetracycline not found in previously available scientific publications.

## Figures and Tables

**Table 1:** Levels of variables on a conditional and natural scale.

Main Components	Factor	Average Level, *q*, wt%	Coefficient of Variation, Δq, wt%	Values of the Levels of Variables (wt%) of Corresponding to Conventional Units		
				−1	0	+1
Oxytetracycline	*x*_1_	1.0	0.5	0.5	1.0	1.5
Nanosilver	*x*_2_	0.075	0.025	0.050	0.075	0.100

**Table 2:** Scheme of experiment planning.

Experiment Number (u)	*x*_0_	*x*_1_	*x*_2_	$x_{3}=x_{1}^{2}-d$	$x_{4}=x_{2}^{2}-d$	$x_{1}x_{2}$
1	1	−1	−1	0.33	0.33	+1
2	1	+1	−1	0.33	0.33	−1
3	1	−1	+1	0.33	0.33	−1
4	1	+1	+1	0.33	0.33	+1
5	1	0	0	−0.67	−0.67	0
6	1	+1	0	0.33	−0.67	0
7	1	−1	0	0.33	−0.67	0
8	1	0	+1	−0.67	0.33	0
9	1	0	−1	−0.67	0.33	0
$\sum_{u-1}^{N}x_{iu}^{2}$	9	6	6	2	2	4

**Table 3:** The results of the research of the flexural strength of composite materials.

The Experiment Number	The Contents of the Component *q*, wt%.		The Flexural Strength, *R_bm_*, MPa	The Flexural Modulus *E*, GPa
	*x*_1_	*x*_2_	*y*	*y*_2_
1	0.5	0.050	92.4	3.1
2	1.5	0.050	82.5	2.7
3	0.5	0.100	80.1	2.8
4	1.5	0.100	83.2	2.8
5	1.0	0.075	81.5	2.9
6	1.5	0.075	87.7	3.3
7	0.5	0.075	84.5	3.0
18	1.0	0.100	80.4	2.7
9	1.0	0.050	82.7	2.8

**Table 4:** Coefficients of the regression equation of the CM destructive stresses.

*b*_0_	*b*_1_	*b*_2_	*b*_11_	*b*_22_	*b*_12_
82.21	−0.60	−2.32	3.53	−1.02	3.25

**Table 5:** Values of adequacy variance ($S_{ui}^{2}$) and reproduced variances ($\sigma^{2}{\left\{y\right\}}_{i}$).

Experiment Number	Adequacy Variance		Reproduced Variances	
	Symbol	Value	Symbol	Value
1	$S_{u1}^{2}$	0.01	$\sigma^{2}{\left\{y\right\}}_{1}$	0.02
2	$S_{u2}^{2}$	0.03	$\sigma^{2}{\left\{y\right\}}_{2}$	0.06
3	$S_{u3}^{2}$	0.01	$\sigma^{2}{\left\{y\right\}}_{3}$	0.02
4	$S_{u4}^{2}$	0.04	$\sigma^{2}{\left\{y\right\}}_{4}$	0.08
5	$S_{u5}^{2}$	0.03	$\sigma^{2}{\left\{y\right\}}_{5}$	0.06
6	$S_{u6}^{2}$	0.04	$\sigma^{2}{\left\{y\right\}}_{6}$	0.08
7	$S_{u7}^{2}$	0.03	$\sigma^{2}{\left\{y\right\}}_{7}$	0.06
8	$S_{u8}^{2}$	0.03	$\sigma^{2}{\left\{y\right\}}_{8}$	0.06
9	$S_{u9}^{2}$	0.01	$\sigma^{2}{\left\{y\right\}}_{9}$	0.02

**Table 6:** Experimental results of the research of the values of the flexural strength during the bending of materials.

Experiment Number	Flexural Strength, *R_bm_*, MPa			Average Value, *R_bm_*, MPa
	1	2	3	
1	92.5	92.3	92.4	92.4
2	82.7	82.4	82.4	82.5
3	80.0	80.2	80.1	80.1
4	83.4	83.2	83.0	83.2
5	81.3	81.6	81.6	81.5
6	87.9	87.5	87.7	87.7
7	84.6	84.6	84.3	84.5
8	80.5	80.5	80.2	80.4
9	82.7	82.8	82.6	82.7

**Table 7:** Calculated values of Student’s *t*-tests (*t_p_*) and dispersion of the regression coefficients ($S_{b_{}}^{2}$).

Experiment Number	Dispersion of the Regression Coefficients		Calculated Values of Student’s *t*-tests	
	Symbol	Value	Symbol	Value
1	$S_{b_{0}}^{2}$	0.003	*t*_0*p*_	1515.96
2	$S_{b_{1}}^{2}$	0.004	*t*_1*p*_	9.19
3	$S_{b_{2}}^{2}$	0.004	*t*_2*p*_	35.50
4	$S_{b_{11}}^{2}$	0.013	*t*_11*p*_	31.26
5	$S_{b_{22}}^{2}$	0.013	*t*_22*p*_	8.99
6	$S_{b_{12}}^{2}$	0.006	*t*_12*p*_	40.7

**Table 8:** Experimental results of the research of the values of the flexural strength during the bending of materials.

*b*_0_	*b*_1_	*b*_2_	*b*_11_	*b*_22_	*b*_12_
2.97	−0.02	−0.05	0.15	−0.25	0.10

**Table 9:** Values of adequacy variance ($S_{ui}^{2}$ ) and reproduced variances (*σ*^2^(*y*)).

Experiment Number	Adequacy Variance		Reproduced Variances	
	Symbol	Value	Symbol	Value
1	$S_{u1}^{2}$	0.003	$\sigma^{2}{\left\{y\right\}}_{1}$	0.005
2	$S_{u2}^{2}$	0.005	$\sigma^{2}{\left\{y\right\}}_{2}$	0.010
3	$S_{u3}^{2}$	0.006	$\sigma^{2}{\left\{y\right\}}_{3}$	0.013
4	$S_{u4}^{2}$	0.003	$\sigma^{2}{\left\{y\right\}}_{4}$	0.005
5	$S_{u5}^{2}$	0.014	$\sigma^{2}{\left\{y\right\}}_{5}$	0.029
6	$S_{u6}^{2}$	0.004	$\sigma^{2}{\left\{y\right\}}_{6}$	0.007
7	$S_{u7}^{2}$	0.020	$\sigma^{2}{\left\{y\right\}}_{7}$	0.039
8	$S_{u8}^{2}$	0.002	$\sigma^{2}{\left\{y\right\}}_{8}$	0.005
9	$S_{u9}^{2}$	0.010	$\sigma^{2}{\left\{y\right\}}_{9}$	0.020

**Table 10:** Experimental results of the research of the values of the flexural modulus of composite materials.

Experiment Number	Flexural Modulus, *E*, GPa			Average Value, *E*, GPa
	1	2	3	
1	3.05	3.10	3.15	3.1
2	2.75	2.73	2.62	2.7
3	2.88	2.80	2.72	2.8
4	2.85	2.80	2.75	2.8
5	2.78	2.90	3.02	2.9
6	3.36	3.30	3.24	3.3
7	3.00	2.86	3.14	3.0
8	2.70	2.75	2.65	2.7
9	2.80	2.90	2.70	2.8

**Table 11:** Calculated values of Student’s *t*-tests (*t_p_*) and dispersion of the regression coefficients ($S_{b_{}}^{2}$ ).

Experiment Number	Dispersion of the Coefficients of the Polynomial Derived		Calculated Values of Student’s *t*-tests	
	Symbol	Value	Symbol	Value
1	$S_{b_{0}}^{2}$	0.001	*t*_0*p*_	99.23
2	$S_{b_{1}}^{2}$	0.001	*t*_1*p*_	0.48
3	$S_{b_{2}}^{2}$	0.001	*t*_2*p*_	1.43
4	$S_{b_{11}}^{2}$	0.004	*t*_11*p*_	2.47
5	$S_{b_{22}}^{2}$	0.004	*t*_22*p*_	4.12
6	$S_{b_{12}}^{2}$	0.002	*t*_12*p*_	2.30

**Table 12:** Composition of composites based on DER-331 epoxy oligomer with different filler contents.

Sequence Number of the Sample	Type of Filler and Its Content in the Epoxy Oligomer DER-331
1	Epoxy matrix DER-331
2	Oxytetracycline of the content *q* = 0.5 wt% (pointed to 100 wt% epoxy DER-331)
3	Oxytetracycline of the content *q* = 1.0 wt%
4	Oxytetracycline of the content *q* = 1.5 wt%
5	Nanosilver of the content *q* = 0.050 wt% (dispersibility 10–100 nm)
6	Nanosilver of the content *q* = 0.075 wt% (dispersibility 10–100 nm)
7	Nanosilver of the content *q* = 0.100 wt% (dispersibility 10–100 nm)
8	Control steel sample without coating (Steel Grade-Fe360-C)
9	Oxytetracycline of the content *q* = 0.5 wt%. + Nanosilver of the content *q* = 0.075 wt%.
10	Oxytetracycline of the content *q* = 0.5 wt%. + Nanosilver of the content *q* = 0.050 wt%.
11	Oxytetracycline of the content *q* = 1.5 wt%. + Nanosilver of the content *q* = 0.075 wt%.

**Table 13:** Results of in vitro tests to assess the safety of nanomaterials in the model system of typical intestinal microflora.

Sequence Number of the Sample	Type of Filler and Its Content in the Epoxy Oligomer DER-331	Initial/Final Number of Test Cultures, lg CFU/mL		
		*Lactobacillus acidophilus*	*Bifidobacterium bifidum*	*Escherichia coli*
2	Control steel sample without coating (Steel Grade-Fe360-C)	2.0/4.0	2.0/2.82	2.0/3.21
3	Oxytetracycline on the content *q* = 0.5 wt% (pointed to 100 wt% epoxy DER-331)	2.0/2.34	2.0/2.34	2.0/2.34
4	Oxytetracycline on the content *q* = 1.0 wt%.	2.0/2.10	2.0/2.11	2.0/2.11
5	Oxytetracycline on the content *q* = 1.5 wt%	2.0/2.11	2.0/1.57	2.0/1.57
6	Nanosilver on the content *q* = 0.050 wt% (dispersibility 10–100 nm)	2.0/2.40	2.0/2.27	2.0/2.27
7	Nanosilver on the content *q* = 0.075 wt% (dispersibility 10–100 nm)	2.0/2.36	2.0/2.43	2.0/2.43
8	Nanosilver on the content *q* = 0.100 wt% (dispersibility 10–100 nm)	2.0/2.35	2.0/2.43	2.0/2.42
9	Oxytetracycline on the content *q* = 0.5 wt% + Nanosilver on the content *q* = 0.075 wt%	2.0/2.08	2.0/2.59	2.0/2.59
10	Oxytetracycline on the content *q* = 0.5 wt% + Nanosilver on the content *q* = 0.050 wt%	2.0/2.48	2.0/2.69	2.0/2.69
11	Oxytetracycline on the content *q* = 1.5 wt% + Nanosilver on the content *q* = 0.075 wt%	2.0/1.89	2.0/1.04	2.0/1.05

## Data Availability

Not applicable.
